# The COMT Genetic Factor Regulates Chemotherapy-Related Prospective Memory Impairment in Survivors With HER2−/+ Breast Cancer

**DOI:** 10.3389/fonc.2022.816923

**Published:** 2022-02-08

**Authors:** Wen Li, Qianqian Zhang, Yinlian Cai, Tingting Chen, Huaidong Cheng

**Affiliations:** Cancer Treatment Center, the Second Affiliated Hospital of Anhui Medical University, Hefei, China

**Keywords:** catechol-O-methyltransferase (COMT), polymorphisms, chemotherapy, memory, human epidermal growth factor receptor 2 (HER2), breast cancer

## Abstract

**Background:**

Previous findings indicated that polymorphism in gene catechol-O-methyltransferase (COMT) had been linked to chemotherapy-related cognitive impairment (CRCI). Nevertheless, the motivation of COMT polymorphisms in regulating cognitive impairment in breast cancer survivors with disparate status of human epidermal growth factor receptor 2 (HER2) was still vague.

**Objective:**

The current research aimed to evaluate the regulation of the risk by COMT genotype on CRCI in breast cancer survivors with disparate status of HER2.

**Methods:**

Breast cancer survivors (103 with HER2− and 118 with HER2+) underwent neuropsychological tests before and after chemotherapy, containing event- and time-based prospective memory (EBPM and TBPM). Three single-nucleotide polymorphisms (SNPs) were estimated by providing peripheral blood, containing COMT (rs165599, rs737865, and rs4680).

**Results:**

The EBPM and TBPM performances was lower as compared with these before chemotherapy (z = −7.712, z = −2.403, respectively, *p* < 0.01). Furthermore, the EBPM and TBPM performances of HER2− group survivors were lower than those of HER2+ group survivors after chemotherapy (z = −7.181, *p* < 0.01; z = −2.205 *p* < 0.05, respectively). The survivors with COMT (rs165599) A/A genotype carriers had a meaningfully poorer chance of memory descend [dominant model: adjusted, OR = 2.21, CI (95%) = 1.156–4.225, *p* = 0.016] and showed better on TBPM test, relative to G/G genotype. Patients with the COMT (rs737865) A/G and G/G genotype showed protective function than the patients with the A/A and performed better on MMSE and TBPM tests.

**Conclusion:**

The types of HER2 may be correlated to chemotherapy-related prospective memory impairments in breast cancer survivors. Furthermore, the COMT (rs165599, rs737865) polymorphisms were correlated to the risk of TBPM decline scores and possibly be a potential genetic identifying for increasing risk of CRCI in breast cancer patients with disparate status of HER2.

## Introduction

Breast cancer is the most familiar malignancy in Chinese women and the sixth main cause of cancer-related death (1). By the end of 2008, 169,452 new breast cancer cases were reported in China; 44,908 cases were related deaths (2). It is reported that 1 in 8–10 women in the United States will suffer from breast cancer during their lifetime (3). The incidence rate of breast cancer increased by about 0.3% every year from 2012 to 2016. On the contrary, the mortality rate decreased year by year, decreasing 40% from 1989 to 2017, which avoided the death of 375,900 breast cancer patients (4). Chemotherapy is one of the most main therapeutic methods for breast cancer; the 5-year survival rate of early breast cancer is close to 90%, which leads to a growing concern about the side effects of chemotherapy treatment (5). In addition to the common clinical side effects such as nausea, vomiting, bone marrow suppression, and hair loss, the impact of chemotherapy on cognitive function has attracted more and more attention in the world (6). A large body of evidence have reported that breast cancer patients experience a moderate to severe degree of cognitive impairment during or after chemotherapy (7–9). These cognitive function deficits involved memory, attention, information processing speed, executive function, and visual space function. This phenomenon is referred to as chemotherapy-related cognitive impairment (CRCI) (10). It is estimated that about 35%–70% of breast cancer patients develop CRCI after chemotherapy, which makes survivors unable to recover from pre-cancer life even after the end of treatment, having significant impact on their daily work and life and greatly reducing their quality of life (QOL) (11).

Prospective memory (PM) is outlined as the ability of remembering to carry out a purpose behavior at a convinced time or place in the future. It not only plays an important role in daily life but also an important part of advanced cognitive activities and is a key factor affecting the recovery of patients’ cognitive function (12). PM was usually fallen into two groups: event-based PM (EBPM) and time-based PM (TBPM). Our previous studies found that patients with breast cancer had PM impairment after chemotherapy, especially significant deficit in EBPM (9).

Breast cancer is a highly heterogeneous malignancy; the most important research direction was concentrated in the field of molecular typing (13). The gene status of human epidermal growth factor receptor 2 (HER2) is important, which is key clinical–pathological characteristic for the prognosis recovery of breast cancer (14). There is an online comment that the main confusion in the CRCI study of breast cancer is its significant heterogeneity, as published in *CA: A Cancer Journal for Clinicians* (15). Our previous research findings simulated that heterogeneity among CRCI in breast cancer survivors with estrogen/progesterone receptor negative (ER−/PR−), showing significant damage on EBPM after chemotherapy (8). There was qualitative research that HER2 was crucial for the construction and maintenance in normal brain tissue (16). HER2 had been shown to be overexpressed in human intracranial tumors, such as gliomas, medulloblastomas, and meningiomas (17). Breast cancer with HER2+ had a higher risk of brain metastasis in comparison to those with the HER2− (18). However, the cognitive function impairment of breast cancer survivors with disparate status of HER2 after chemotherapy was still unclear.

Previous studies made known that COMT (rs4680, rs65599, and rs737865) was closely related to cognitive function (19). COMT gene played an important role in memory, executive control, response suppression, reward processing, decision analysis, and other cognitive processes through the regulation of dopaminergic concentration in the human brain (20, 21). Small et al. showed that breast cancer survivors who were COMT Val carriers were susceptible to cognitive deficits following chemotherapy (22). Furthermore, our previous studies had found that COMT (rs165599) gene was associated with retrospective memory (RM) in triple-negative breast cancer (TNBC) survivors (23). Recently, we found that COMT (rs737865) was correlated to EBPM in breast cancer patients with different hormonal receptor expression (24). However, the correlation between the chemotherapy-related PM impairment and the COMT polymorphisms in breast cancer patients with the disparate status of HER2 had not yet been illustrated.

In the current research, we attempt to survey the chemotherapy-related PM impairment in breast cancer survivors with different HER2 and clear cut the genetic features of COMT polymorphisms on CRCI in breast cancer patients with the disparate status of HER2 (HER2−, HER2+).

## Materials and Methods

### Participants

A total of 221 breast cancer patients, who were recruited from 2014 to 2017 in the Department of Oncology, the Affiliated Second Hospital of Anhui Medical University, were assigned to HER2− (103 cases) or HER2+ group (118 cases).

The Research Ethics Committee of the Second Affiliated Hospital of Anhui Medical University, China, approved the research. Written informed consents were obtained from all participants before the research was conducted. Epidemiological data and blood samples were collected in accordance with ethical regulations.

All participants had exceeded 5 years of education and were all right handed. Inclusion criteria were as follows: (1) breast cancer was defined by immunohistochemical and pathological diagnosis and that positive of Her-2 was recorded as standard immunohistochemistry 3+ or ISH positive; (2) adriamycin, paclitaxel, cyclophosphamide, and fluorouracil were applied by standard chemotherapy regimen or combined with Herceptin-targeted therapy, based on chemotherapy, but no hormone therapy; (3) age and pathological type were not limited; (4) the participant could carry out normal daily activities, with Karnofsky performance status scale (KPS) scores ≥80; and (5) there are no communication barriers and could proceed with normal language communication. Exclusion criteria were as follows: (1) a history of radiotherapy and endocrine therapy; (2) advanced cachexia; (3) metastatic encephaloma according to brain imaging examination; (4) anxiety, depression, paranoia, and other mental disorders; (5) medical history of alcohol or psychotropic drug dependence; and (6) clinical diagnose of dementia.

### General Assessment of Cognitive

In accordance with the upward grouping of breast cancer survivors, a battery of cognitive tasks were performed within 1 month before chemotherapy and after six cycles of standard postoperative adjuvant chemotherapy. Mini-mental state examination (MMSE) was used to evaluate general cognitive functioning and the degree of intellect, containing seven aspects, presenting in time orientation, place orientation, immediate memory, attention and computational power, delayed memory, language, and visual space. The verbal fluency test (VFT) was reflected in the patient’s ability to invoke certain kinds of things from the memory base, mainly measuring the ability of spontaneous language movement, where participants were required to speak out as many targets as they could remember in 1 min. The digit span test (DST) was applied to test patients’ short-term memory, including in order and inverted order tests. Participants were required to reiterate the numbers by reading them out to the researchers. The total score corresponded to the number of the last correct character string retelling from the subjects.

### Event-Based Prospective Memory Task

On each card of the 32 cards used, 12 high-frequency Chinese words were printed, of which 10 of 12 words belong to the first category (large category) and the spare two words belong to the second category (animal category). In the learning stage, the participants were required to say the two words pertaining to the small category that differed from the other 10 words on each card. The first two cards were for learning; the first card did not contain the target word, while the second one did. According to the instructions from the experimenter before the test, the target events for the PM task occurred on the 2th (exercise card), 6th, 11th, 16th, 21th, 25th, and 31th card, and each correct score was 1 point; all had 6 points. When the selected word was the target word (animal category), the participants were instructed to tap at the table. At the termination of card selection, they completed another task, that is, let the participants remember to leave their contact number (counted as 2 points). The highest scores of the event-based prospective memory (EBPM) tasks were 8 points.

### Time-Based Prospective Memory Task

On each card of the 100 cards, 12 different two-digit numbers were printed. In the learning stage, participants were required to name the smallest and the largest numbers on each card. According to the instructions from experimenters before the task, when a specific goal time (i.e., at the time points of 5, 10, and 15 min after the beginning of the task), the participants were instructed to knock on the table: 2 points were endowed for responding within 10 s before and 10 s after each target time, and 1 point was endowed for responding within 30 s before and 30 s after each target time, with a topmost score of 6 points. The participant was told that the time can be checked through the clock placed 1 m away behind the subject’s right shoulder. The clock was set to 0:0:0 at the beginning of the experiment, and the task was stopped when the clock indicated 17 min. The maximum score of time-based prospective memory (TBPM) was 6 points.

### Genotyping

The peripheral blood (3–5 ml) of the subject was sampled into the sodium citrate anticoagulation blood tubes and reserved in the refrigerator at −80°C. Genomic DNA was picked up from the peripheral blood with blood genomic DNA Qiagen Kit (Shanghai Genesky Biotechnology Co., Ltd., http://biotech.geneskies.com), operated according to the instructions, and the extracted DNA was stored at −20°C. Genotyping was completed by Shanghai Genesky Biotechnology Co., Ltd. (Shanghai, China), utilizing the improved multiplex ligase detection reaction (iMLDR) technology. Different fluorescently labeled allele-specific oligonucleotide probe pairs were used to identify each SNP allele with high specificity. Nonspecific sequences of different lengths were introduced into the end of the ligation probe, and the ligation products were obtained by ligase chain reaction corresponding to the site. Then, the ligation products were amplified by PCR with fluorescent-labeled universal primers. The PCR-amplified products were separated by fluorescence capillary electrophoresis. Finally, GeneMapper 4.1 (Applied Biosystems, USA) was used to analyze the electropherogram; the genotyping success rate of each SNP locus was obtained. A sample accounting for 10% of the total DNA samples was randomly selected for duplicate tests for quality control.

### Statistical Analysis

Statistical analysis was performed with a one-way ANOVA using SPSS software package (version 22.0, http://spss.en.softonic.com/; Chicago, IL, USA).The basic clinical data and neuropsychological tasks scores were compared between HER2− and HER2+ group. The two independent samples t-test and Mann–Whitney U-test were performed, respectively, for normal and non-normal distribution in continuous variable data. All results are presented in the forms of mean ± standard deviation (SD). Hardy–Weinberg equilibrium (HEW) was applied to analyze whether the distribution of genotype frequency of SNP loci conforms the genetic balance in two groups. In addition, the chi-square (χ^2^) test was used to analyze the differences in alleles, genotype frequency, and other taxonomic variables between the two groups. Logistic regression was reported as the relative risk, odds ratio (OR), and 95% confidence interval (CI), evaluating the susceptible factors of cognitive impairment; a general genetic model (co-dominant, dominant, recessive, and additive models) to single SNP construes was covered, rectifying age, KPS, chemotherapy regimen, level of education, and pathological pattern. Binary logistic regression was applied to analyze the associations between COMT (rs165599 and rs737865) polymorphism and CRCI. A one-way ANOVA was used to analyze the cognitive differences among different genotypes and genetic model (dominant and recessive models). All statistical results were two-tailed probability proofs, and the statistically significant standard was defined at *p* < 0.05.

## Results

### The Basic Clinical Data for Research Objects

**Table 1** has a total of 221 patients conformed to the inclusion criteria; among them, HER2− group included 103 patients, and HER2+ group included 118 patients. There was no striking difference in age (49.02 ± 10.95 vs. 48.56 ± 10.45), level of education (10.09 ± 3.63 vs. 10.10 ± 3.67), and KPS (82.91 ± 8.12 vs. 84.07 ± 7.76). Similarly, no significant differences were found for pathological patterns and cancer stages. In the HER2− group, 95 breast cancer patients were discriminated as non-special-type invasive ductal carcinoma (IDO-NOS), 3 breast cancer patients were discriminated as special-type invasive ductal carcinoma (IDO-S), and 5 patients were discriminated as microinvasive carcinoma (MIC). Similarly, in the HER2+ group, 112 breast cancer patients were discriminated as IDO-NOS, 1 breast cancer patients was identified as carcinoma *in situ* (CIS), and 5 breast cancer patient was identified as MIC. The percentages of stage I (3.9% and 5.9%, respectively) and stage II (52.4% and 48.3%, respectively) were found in breast cancer patients for the two groups. There was significant difference in chemotherapy regimen between the two groups (χ^2^ = 32.101, *p <*0.01). The utilization rate of Trastuzumab accounted for about 23.7% in the HER2+ group.

**Table 1:** The basic clinical dates of breast cancer patients with HER2− and HER2+.

Items		Groups	
		A (n=103)	B (n=118)
Age (mean ± SD, year)		49.02 ± 10.95	48.56 ± 10.45
Education (mean ± SD, year)		10.09 ± 3.63	10.10 ± 3.67
KPS (mean ± SD, year)		82.91 ± 8.12	84.07 ± 7.76
Pathological patterns (%)	IDC-NOS	95 (92.2%)	112 (94.9%)
	IDC-S	3 (2.9%)	0
	CIS	0	1 (0.8%)
	MIC	5 (4.9%)	5 (4.2%)
Stages (%)	I	4 (3.9%)	7 (5.9%)
	II	54 (52.4%)	57 (48.3%)
	III	22 (21.4%)	18 (15.3%)
	IV	23 (22.3%)	36 (30.5%)
Chemotherapy regimen	PTX	6 (5.8%)	13 (11.0%)**
	Trastuzumab + chemotherapy	0	19 (16.1%)
	ADM	22 (21.4%)	28 (23.7%)
	PTX+ADM	75 (72.8%)	58 (49.2%)

**<0.01.

KPS, Karnofsky performance status scale; IDC-NOS, non-special-type invasive ductal carcinoma of breast; IDC-S, special-type invasive ductal carcinoma of breast; CIS, carcinoma in situ; MIC, microinvasive carcinoma; PTX, pacliaxcl; ADM, adriamycin.

### General Assessment of Cognitive, EBPM, and TBPM Scores: Before and After Chemotherapy

**Table 2** reveals that the MMSE was significantly lessened to 26.67 ± 1.64 after chemotherapy in comparison to that before chemotherapy (27.21 ± 1.59, *p* < 0.01). DST and VFT scores were also strikingly lessened from before (6.21 ± 0.71 and 11.43 ± 1.53, respectively) to after (5.79 ± 0.99, *p* < 0.01 and 9.93 ± 2.14, *p* < 0.01, respectively) chemotherapy. The EBPM and TBPM scores were significantly decreased after chemotherapy and manifested as 2.72 ± 0.98 vs. 1.84 ± 1.06 (*p* < 0.01), 4.95 ± 1.03 vs. 4.75 ± 0.92 (*p* < 0.05) and had a significant difference.

**Table 2:** General assessment of cognitive before and after chemotherapy.

Task	Mean ± SD	
	Before chemotherapy (n=221)	After chemotherapy (n=221)
MMSE	27.21 ± 1.59	26.67 ± 1.64**
DST	6.21 ± 0.71	5.79 ± 0.99**
VFT	11.43 ± 1.53	9.93 ± 2.14**
EBPM	2.72 ± 0.98	1.84 ± 1.06**
TBPM	4.95 ± 1.03	4.75 ± 0.92*

*p < 0.05, **p < 0.01.

MMSE, mini-mental state examination; DST, digit span test; VFT, verbal fluency test; EBPM, event-based prospective memory; TBPM, time-based prospective memory.

### General Assessment of Cognitive, EBPM, and TBPM Scores: After Chemotherapy

**Table 3** indicates the MMSE and TBPM scores of breast cancer patients in the HER2+ group after chemotherapy was raised (HER2−: 26.43 ± 1.65 vs. 4.62 ± 0.83; HER2+: 26.89 ± 1.60 vs.4.86 ± 0.98, p < 0.05). Significantly, the DST, VFT, and EBPM were raised in the HER2+ group and manifested as DST of 5.44 ± 0.97 vs. 6.09 ± 0.90, VFT of 9.10 ± 2.14 vs. 10.65 ± 1.86, and EBPM of 1.29 ± 1.13 vs. 2.32 ± 0.72 and had a significant difference (*p* < 0.01).

**Table 3:** General assessment of cognitive in HER2− and HER2+ groups after chemotherapy.

Task	Groups (mean ± SD)	
	Her2− (n=103)	Her2+ (n=118)
MMSE	26.43 ± 1.65	26.89 ± 1.60*
DST	5.44 ± 0.97	6.09 ± 0.90**
VFT	9.10 ± 2.14	10.65 ± 1.86**
EBPM	1.29 ± 1.13	2.32 ± 0.72**
TBPM	4.62± 0.83	4.86 ± 0.98*

*p < 0.05, **p < 0.01.

MMSE, mini-mental state; DST, digit span test; VFT, verbal fluency test; EBPM, event-based prospective memory; TBPM, time-based prospective memory.

### The Unit SNP Loci Analytical Results

The three SNPs of the COMT gene all conformed to Hardy–Weinberg equilibrium (HWE) for the two groups (*p* > 0.05). It indicated the SNP loci gene frequency distribution we chose from large randomly mating population.

**Table 4** shows that the allelic distribution of COMT (rs165599 G vs. A; rs737865 A vs. G) were strikingly different between HER2− and HER2+ survivors (*p* = 0.045, *p* = 0.012, respectively). In **Table 5**, COMT rs165599 (co-dominant model: χ^2^ = 6.909, *p* = 0.032; dominant model: χ^2^ = 6.042, *p* = 0.014) and rs737865 (co-dominant model: χ^2^ = 10.993, *p* = 0.004; dominant model: χ^2^ = 4.766, *p* = 0.029; recessive model: χ^2^ = 7.418, *p* = 0.006) genotypic frequency distribution acted out strikingly different. Besides, logistic regression analysis results revealed that COMT rs165599 G/A genotypes [rectified, OR = 0.399, CI (95%) = 0.174–0.918, *p* = 0.031] had strikingly reduced occurrences of expanding cognitive descend than the patients with G/G. For the genetic model, the dominant model of rs165599 with G/A and A/A genotype [rectified, OR = 2.21, CI (95%) = 1.156–4.225, *p* = 0.016] could reduce the risk of cognitive decline. The A/G and G/G [rectified, OR = 0.178, CI (95%) = 0.054–0.579, *p* = 0.004; OR = 0.285, CI (95%) = 0.086–0.947, *p* =0.040, respectively] genotype of the COMT rs737865 had strikingly lower odds of expanding cognitive descend than the patients with the A/A genotype. The rs737865 was discovered to strikingly enhance the venture of CRCI in the dominant model [rectified, OR = 1.999, CI (95%) = 1.139–3.509, *p* = 0.016] and recessive model [rectified, OR = 4.595, CI (95%) = 1.453–14.532, *p* = 0.009].When comparing the addictive models [rectified, OR = 0.769, CI (95%) = 0.450–1.408, *p* = 0.433], no significant correlations were established for COMT rs737865. There was no statistically striking difference in the locus of COMT rs4680 between the HER2− and HER2+ group.

**Table 4:** Information about three genotyped SNPs loci of COMT gene in HER2− and HER2+ groups .

SNP	COMT		
	rs4680	rs165599	rs737865
**CHR**	22	22	22
**Allele position**	19951271	19956781	19930121
**Ref allele**	G	G	A
**Alt allele**	A	A	G
**MAF**	0.233	0.422	0.226
**P for HWE**	0.279	0.227	0.261
p*	0.648	0.045	0.012

SNP, single-nucleotide polymorphism; CHR, chromosome; Ref allele, loci alleles on the reference sequence; Alt allele, the other allele on the loci; MAF, minor allele frequency (data from 1000 Genomes); HWE, Hardy–Weinberg equilibrium, p-value for HWE in two groups.

^*^p-value for allele frequency differences between two groups.

**Table 5:** Genotype frequencies of SNPs of the COMT (rs4680, rs165599, and rs737865) genes between two groups.

SNP	Model	Genotype	Her2 (−)	Her2 (+)	*p* ^a^ (χ^2^)	Logistic regression	
						OR (95%CI)	*p* ^b^
rs4680	Co-dominant	G/G	63	65		–	–
		G/A	32	44	0.615	1.192 (0.409-3.478)	0.747
		A/A	8	9		1.597 (0.524-4.865)	0.410
	Dominant	G/A+A/A	40	53	0.361	1.233 (0.706-2.153)	0.461
		G/G	63	65			
	Recessive	A/A	8	9	0.969	0.752 (0.263-2.147)	0.594
		G/G+G/A	95	109			
	Addictive	–	–	–	–	0.732 (0.409-1.308)	0.292
rs165599	Co-dominant	G/G	33	21		–	–
		G/A	53	67	0.032	0.399 (0.174-0.918)	0.031
		A/A	17	30		0.84 (0.407-1.736)	0.638
	Dominant	G/A+A/A	70	97	0.014	2.21 (1.156-4.225)	0.016
		G/G	33	21			
	Recessive	A/A	17	30	0.106	1.511 (0.758-3.014)	0.241
		G/G+G/A	86	88			
	Addictive	–	–	–	–	0.72 (0.413-1.255)	0.246
rs737865	Co-dominant	A/A	60	52		–	–
		A/G	38	47	0.004	0.178 (0.054-0.579)	0.004
		G/G	4	19		0.285 (0.086-0.947)	0.040
	Dominant	A/G+G/G	42	66	0.029	1.999 (1.139-3.509)	0.016
		A/A	60	52			
	Recessive	G/G	4	19	0.006	4.595 (1.453-14.532)	0.009
		A/A+A/G	98	99			
	Addictive	–	–	–	–	0.769 (0.450-1.408)	0.433

a The χ^2^ test of p-values for SNP polymorphisms distribution differences between Her2(−) and Her2(+) group.

b p-value for logistic regression analysis; odds ratio (the OR); 95% confidence interval (95%CI); models: various genetic models that were defined as 1 (MM + Mm) versus 0 (mm) for dominant; 1 (mm) versus 0 (MM + Mm) for recessive; and 0 (mm) versus 1 (Mm) versus 2 (MM) for additive and co-dominant (M and m represent major and minor alleles, respectively).

### The Correlation Analysis Between COMT (rs165599 and rs737865) Gene Polymorphisms and CRCI

As **Table 6** shows, the A/A genotype carriers of COMT rs165599 showed distinctly elevated scores on TBPM (4.94 ± 0.75 vs. 4.42 ± 0.71, *p* < 0.05) than G/G carriers in breast cancer patients with disparate status of HER2. Similarly, the G/G and A/G genotype carriers of COMT rs737865 represented higher scores on MMSE (HOM: 24.50 ± 2.38 vs. 26.88 ± 1.26, *p* < 0.01; HET: 25.87 ± 1.85 vs. 26.88 ± 1.26, *p* < 0.01, respectively) tests and TBPM (dominant model: 4.88 ± 0.71 vs. 4.45 ± 0.87, *p* < 0.01; HET: 4.89 ± 0.69 vs. 4.45 ± 0.87, *p* < 0.01, respectively) tests than A/A carriers.

**Table 6:** Comparison for neuropsychological performance of COMT (rs165599 and rs737865) genotypes and genetic model.

rs165599	Dominant		Recessive		HOM		HET	
	G/A+A/A vs. G/G		A/A vs. G/G+G/A		A/A vs. G/G		G/A vs. G/G	
MMSE	26.27 ± 1.72	26.76 ± 1.48	26.29 ± 1.65	26.45 ± 1.66	26.29 ± 1.65	26.76 ± 1.48	26.26 ± 1.76	26.76 ± 1.48
DST	5.42 ± 0.97	5.49 ± 0.98	5.56 ± 1.03	5.42 ± 0.96	5.56 ± 1.03	5.49 ± 0.98	5.38 ± 0.95	5.49 ± 0.98
VFT	8.87 ± 2.14	9.58 ± 2.09	9.35 ± 2.03	9.05 ± 2.17	9.35 ± 2.03	9.58 ± 2.09	8.72 ± 2.17	9.58 ± 2.09
EBPM	1.34 ± 1.17	1.18 ± 1.04	1.71 ± 1.36	1.21 ± 1.06	1.71 ± 1.36	1.18 ± 1.04	1.23 ± 1.09	1.18 ± 1.04
TBPM	4.71 ± 0.87	4.42 ± 0.71	4.94 ± 0.75	4.56 ± 0.84	4.94 ± 0.75	4.42 ± 0.71*	4.64 ± 0.90	4.42 ± 0.71
**rs737865**	**Dominant**		**Recessive**		**HOM**		**HET**	
	**A/G+G/G vs. A/A**		**G/G vs. A/A+A/G**		**G/G vs. A/A**		**A/G vs. A/A**	
MMSE	25.74 ± 1.91	26.88 ± 1.26**	24.50 ± 2.38	26.49 ± 1.59*	24.50 ± 2.38	26.88 ± 1.26**	25.87 ± 1.85	26.88 ± 1.26**
DST	5.52 ± 1.04	5.39 ± 0.92	5.25 ± 0.96	5.45 ± 0.97	5.25 ± 0.96	5.39 ± 0.92	5.55 ± 1.06	5.39 ± 0.92
VFT	8.93 ± 1.92	9.25 ± 2.29	8.75 ± 2.75	9.13 ± 2.13	8.75 ± 2.75	9.25 ± 2.29	8.89 ± 1.86	9.25 ± 2.29
EBPM	1.12 ± 1.09	1.43 ± 1.14	1.75 ± 1.50	1.29 ± 1.11	1.75 ± 1.50	1.43 ± 1.14	1.05 ± 1.04	1.43 ± 1.14
TBPM	4.88 ± 0.71	4.45 ± 0.87**	4.75 ± 0.96	4.62 ± 0.83	4.75 ± 0.96	4.45 ± 0.87	4.89 ± 0.69	4.45 ± 0.87**

*p < 0.05; **p < 0.01.

MMSE, mini-mental state; DST, digit span test; VFT, verbal fluency test; EBPM, event-based prospective memory; TBPM, time-based prospective memory.

Models: Various genetic models that were defined as 1 (MM + Mm) versus 0 (mm) for dominant; 1 (mm) versus 0 (MM + Mm) for recessive; homozygote (HOM); heterozygote (HET).

## Discussion

The results of the current study revealed that, first, breast cancer survivors after chemotherapy had memory impairment on EBPM and TBPM compared to that before chemotherapy. Second, breast cancer patients with HER2− have poorer MMSE, DST, VFT, TBPM, and EBPM scores after chemotherapy than that of patients with HER+. Third, there were significant differences on genotypes about COMT (rs165599 and rs737865) between HER− and HER+ groups; the A/A carriers of COMT rs165599 and the G/G and A/G carriers of COMT rs737865 performed more poorly than COMT (G/G, A/A, respectively) carriers on tests of TBPM in breast cancer patients with HER2−, and the COMT polymorphism may be an underlying genetic factor for the enhancement of the venture to chemotherapy-related PM impairment in breast cancer patients with disparate status of HER2. The results of this study are innovative in that they represent the first demonstration of a link between a risk factor for CRCI and COMT genotype in breast cancer patients with the disparate status of HER2.

Cancer patients will have a series of cognitive changes after chemotherapy, among which memory impairment was one of the main performances (25). Kanaskie et al. (26) believes that the changes in cognitive function are the side effects after chemotherapy for some breast cancer survivors, which include subtle changes in memory, attention, and executive function. Ibrahim et al. (27) found that Taxane-based cognitive impairment is more common in the areas of attention, executive function, and depression, and visual memory in breast cancer patients at 6 months or more after treatment. Andryszak et al. found that anthracycline-based adjuvant chemotherapy (AC) was associated with delayed memory deficits after chemotherapy, and about 19% of breast cancer patients deteriorated after treatment (28). Our previous study found that breast cancer patients mainly present with PM impairment after chemotherapy, especially EBPM deficits (9). Further research found that breast cancer patients with ER−/PR− performed worse on EBPM than those with ER+/PR+ after chemotherapy (8). In this study, 221 breast cancer patients were found to have a decline in cognitive function following chemotherapy, and in breast cancer patients with disparate expression of HER2, there exists an obvious difference in EBPM and TBPM after chemotherapy.

HER2 is a proto-oncogene, which can lead to resistance to tumor cells apoptosis and the proliferation tumor blood vessels and lymphatic vessels (29). HER2 was a prognostic factor, which was closely related to recurrence-free survival and overall survival; approximately 18–30% breast cancer patients shows high expression of HER2 (30). HER2-positive breast cancer patients can be assigned to luminal B (endocrine therapy responsive) or HER2 enriched (endocrine therapy unresponsive), according to their molecular subtypes (31). The combination of trastuzumab (the most widely used anti-HER2 drug) with chemotherapy resulted in significant improvement in the poor prognosis of early HER2+ breast cancer patients and reduced the recurrence risk and the mortality (32). With the application of trastuzumab, about 85% of HER2+ breast cancer patients were expected to survive for at least 10 years, and the prognosis of these patients has improved dramatically (33). Trastuzumab can prolong the survival time of breast cancer patients, but the research on the effect of anti-HER2 therapy on cognitive function is very rare and controversial, and the findings on the correlation between HER2 status and cognitive deficits are full of contradictions. Some studies showed that there was no correlation between cancer HER2 status and pre-adjuvant therapy cognitive impairment in elderly breast cancer (>65 years of age) (34). On the contrary, Koleck et al. (35) found that the HER2-positive breast cancer patients were more likely to get poorer verbal, visual, and visual working memory performance compared to HER2-negative patients before adjuvant chemotherapy. One study found that the slight to significant deterioration of cognitive function was reported in breast cancer treatment following chemotherapy regimens containing trastuzumab (36). Lee et al. identified that chemo-brain was induced after trastuzumab treatment in an HER2-positive gastric cancer model, and atorvastatin could improve the cognitive impairment caused by trastuzumab (37). However, there were also findings indicating that the administration of subcutaneous trastuzumab can reduce the symptoms of nausea and vomiting caused by chemotherapy and had no negative impact on health-related quality of life (38). The incidence of suspected mild cognitive impairment was 28.6% in the trastuzumab plus chemotherapy group. It showed slightly better cognitive function than that with trastuzumab mono-therapy in HER2+ breast cancer (39). Until now, there is no report regarding CRCI in breast cancer with anti-HER2 therapy. In this study, breast cancer patients with HER2− have a more significant damage on neuropsychological tasks than patients with HER2+. This may be due to the improvement of cognitive function in patients with trastuzumab combined with chemotherapy. In the HER2− group, TNBC patients accounted 78.64%; the CRCI of this group was strikingly higher than that of the HER2+ group, which was consistent with our previous research (23).

The COMT gene was expressed throughout the brain, and its translation products played a key role in clearing catecholamines, such as dopamine, epinephrine, and norepinephrine (40). The expression level and product of COMT gene are affected by many factors. Breast cancer is a tumor closely associated with estrogen, and estrogen could downregulate the level of COMT gene, decreasing the activity of COMT enzyme (41). It has been found that estrogen inhibits COMT gene transcription *via* promoter reporter gene (42). Estrogen enhanced the promoters of DNMT3B, MBD2, and HDAC1 in breast cancer cells and reduced COMT transcription, resulting in increased DNA oxidative damage (43). Catecholestrogens were estradiol and estrone metabolites produced in breast cancer cells, and its derivatives could initiate estrogen receptor-mediated processes (44). The expression of COMT mRNA and protein was decreased by the proinflammatory cytokine tumor necrosis factor alpha (TNFα) in astrocytes, and neuroinflammation could be found in the recovery phase (45). There were at least eight different SNPs loci obtained on COMT gene, among which Val158Met locus had been studied the most frequently (46). COMT polymorphisms are manifested as a valine (Val or G) and methionine (Met or A) mutation at codon 158. The activity of the COMT enzyme with the Met carriers was three- to fourfold reduced than that with the Val carriers, increasing the dopaminergic concentration of synapses in the human brain (47). McIntosh et al. (48) found that the anterior cingulate cortex of Val homozygous carriers was significantly smaller than that of met carriers in schizophrenic patients; the altered brain structure could lead to cognitive impairment. COMT gene was widely expressed in the hippocampus and was associated with memory function (19, 49). Correa et al. (50) found that COMT SNPs were strikingly associated with attention, executive functions, and memory scores in patients with brain tumor. Matsuzaka et al. (51) showed the relationship between the two SNPs of the COMT (rs165599 and rs737865) and working memory; the cognition in schizophrenia patients may be modulated by COMT. Compared with healthy controls, breast cancer patients receiving chemotherapy had slower treatment speed and poorer executive function, while apolipoprotein E (APOE) and COMT gene polymorphisms were associated with cognitive impairment (52).Our previous research findings indicated that COMT (rs165599) was a risk-related genetic factor influencing CRCI in TNBC patients (23). Further study found that COMT (rs737865) was correlated with EBPM damage following chemotherapy in breast cancer with different expressions of hormone receptor (24). In this study, the A/A genotype carriers of COMT (rs165599) and G/G genotype carriers of COMT (rs737865) had higher scores on TBPM after chemotherapy and were genetic risks for CRCI in breast cancer with disparate expression of HER2.

Finally, limitations of this research should be recognized. First, this research only compared the changes in cognitive function in breast cancer patients with disparate expressions of HER2 before and after chemotherapy, lacking a healthy control group. Second, follow-up study was lacking. Cognitive impairments following chemotherapy may change in the later follow-up period. Thus, further research is needed. Third, the results were subjective memory impairment; further objective cognitive tests need to be clarified in future research. Fourth are the impacts of chemotherapy regimens. If some regimens had negative effects on cognitive function, but others did not, the effects of the former would be diluted and undetectable. Finally, the sample size in this research was small, and the numbers of breast cancer patients were scarce, therefore needing further supplement.

In a word, our study preliminarily found some differences in chemotherapy-related PM impairment and genetic polymorphisms in breast cancer patients with the disparate HER2. The heterogeneity of CRCI may be rectified by COMT (rs165599, rs737865) polymorphism, and this rectification may possibly show that COMT polymorphism is a risk leading to a lower memory performance in breast cancer patients with disparate HER2.

## Conclusion

In brief, we conducted the discrepancy between chemotherapy-related PM impairment and genetic polymorphisms in patients with HER2−/+ breast cancer. The consequences indicated that the heterogeneity of CRCI may be regulated by COMT (rs165599 and rs737865), which may affect the CRCI in breast cancer with disparate status of HER2.

## Data Availability Statement

The datasets presented in this study can be found in online repositories. The names of the repository/repositories and accession number(s) can be found below: https://www.ncbi.nlm.nih.gov/, 111.

## Ethics Statement

The studies involving human participants were reviewed and approved by the Research Ethics Committee of the Second Affiliated Hospital of Anhui Medical University. The patients/participants provided their written informed consent to participate in this study.

## Author Contributions

WL performed data collection, cognitive tests, EBPM and TBPM task, and blood collection. QZ and YC performed statistical analysis. TC performed data acquisition. HC designed the project and wrote the manuscript. All authors contributed to the article and approved the submitted version.

## Conflict of Interest

The authors declare that the research was conducted in the absence of any commercial or financial relationships that could be construed as a potential conflict of interest.

## Publisher’s Note

All claims expressed in this article are solely those of the authors and do not necessarily represent those of their affiliated organizations, or those of the publisher, the editors and the reviewers. Any product that may be evaluated in this article, or claim that may be made by its manufacturer, is not guaranteed or endorsed by the publisher.
